# Comparative Study of Alternative Methods for Measuring Leg Length Discrepancy after Robot-Assisted Total Hip Arthroplasty

**DOI:** 10.3390/bioengineering11080853

**Published:** 2024-08-21

**Authors:** Hamad Nazmy, Giovanni Solitro, Benjamin Domb, Farid Amirouche

**Affiliations:** 1 Department of Orthopaedic Surgery, University of Illinois at Chicago, Chicago, IL 60612, USA; hamad2@uic.edu; 2 Department of Orthopaedics, LSU Health Shreveport, Shreveport, LA 71103, USA; solitrogf@hotmail.com; 3 American Hip Institute, 999 E. Touhy, Suite 450, Des Plaines, IL 60018, USA; bendomb@gmail.com; 4 Orthopaedic and Spine Institute, Department of Orthopaedic Surgery, Northshore University Health System, an Affiliate of the University of Chicago Pritzker School of Medicine, 9669 Kenton Avenue, Skokie, IL 60076, USA

**Keywords:** leg length discrepancy, total hip arthroplasty, pelvic obliquity, robot-assisted THA, femoral misalignment

## Abstract

Background: Our study addresses the lack of consensus on measuring leg length discrepancy (LLD) after total hip arthroplasty (THA). We will assess the inter-observer variability and correlation between the five most commonly used LLD methods and investigate the use of trigonometric principles in overcoming the limitations of current techniques. Methods: LLD was measured on postoperative AP pelvic radiographs using five conventional methods. CT images created a 3D computer model of the pelvis and femur. The resulting models were projected onto a 2D, used to measure LLD by the five methods. The measurements were evaluated via Taguchi analysis, a statistical method identifying the process’s most influential factors. The approach was used to assess the new trigonometric method. Results: Conventional methods demonstrated poor correlation. Methods referenced to the centers of the femoral heads were insensitive to LLD originating outside the acetabular cup. Methods referencing either the inter-ischial line or the inter-obturator foramina to the lesser trochanter were sensitive to acetabular and femoral components. Trigonometry-based measurements showed a higher correlation. Conclusions: Our results underscore clinicians’ need to specify the methods used to assess LLD. Applying trigonometric principles was shown to be accurate and reliable, but it was contingent on proper radiographic alignment.

## 1. Introduction

Total hip arthroplasty (THA) is recognized as a successful and reliable intervention, usually performed to relieve pain or improve hip mobility and stability [1,2]. Obtaining the correct center of rotation, the orientation of implant components, appropriate femoral offset, and equal leg lengths are critical in restoring optimal hip biomechanics through this procedure [3,4,5,6].

In the era of increased healthcare services marketing, patient satisfaction is often used as a surrogate to gauge the overall success of medical practice [7]. However, typical patient satisfaction rates for primary THA have historically been greater than 90% [8]. Recent multi-center studies using validated tools continue to identify that 8–11% of patients who undergo THA continue to report postoperative dissatisfaction [7,9,10,11]. Postoperative LLD is often the basis for this dissatisfaction and has been found to occur in up to 26% of THA cases [4,12]. Data published by the Joint Commission on Accreditation of Healthcare Organizations (JCAHO) reveals that LLD may account for as much as 4.7% of all medical errors and contribute significantly to increased patient morbidity [13]. Therefore, it is unsurprising that LLD following THA is a leading cause of litigation against orthopedic surgeons [14].

The precise boundary between acceptable and unacceptable leg length discrepancy (LLD) levels remains unclear [15,16,17]. However, it is widely agreed that LLD magnitudes exceeding 1.5 cm can have adverse effects on patients, including sciatic, femoral, or peroneal nerve palsies, chronic lower back pain, hip dislocation, the need for a shoe lift, or gait disorders [18]. Studies have also shown a link between higher rates of postoperative trochanteric pain and suspected aseptic loosening with significant LLD. Additionally, compensatory gait abnormalities resulting from underlying LLD can lead to degenerative arthritis of the lower extremities and lumbar spine [19,20]. These factors likely contribute to overall patient dissatisfaction [13].

When assessing LLD, the discrepancy must be determined to be true (anatomical) or apparent (functional) [21]. Factual discrepancies exist in which an actual bony asymmetry exists between the head of the femur and the ankle mortise. Functional LLDs occur as physiological responses to altered biomechanics and could be caused by soft-tissue contractures, spinal deformities, or other processes that lead to pelvic obliquity. Recognition of functional LLD is essential as this is what the patient perceives. Recent studies have demonstrated that apparent LDD, not anatomical LLD, better predicts poor physical performance and reduced mobility after THA [4,21,22,23,24]. In light of this, the American Academy of Orthopedic Surgeons recommends that in addition to measuring actual and apparent limb lengths before performing THA—abduction, adduction, and flexion contractures should be assessed and quantified. Clinically, most discrepancies result from actual and apparent differences [25,26].

Clinical assessment of true limb length can be conducted by measuring the distance between the anterior superior iliac spine (ASIS) and the medial malleolus. Similarly, apparent length can be measured from the xiphisternum or umbilicus to the medial malleoli of the ankle. Of these two “direct” methods, the measurement of true length is arguably more reliable than apparent length [25]. However, neither method comprehensively assesses the effects of soft-tissue contractures or pelvic obliquity. A more reliable clinical technique for measuring LLD is to level the pelvis of a standing patient by placing blocks of known height under their shorter limb. This is called the “indirect” method [23,26]. Although these clinical methods are easy, safe, and non-invasive means of assessing limb length, they are comparatively less precise and reliable than radiographic techniques. Studies have also found that clinical techniques correlate poorly with LLD measurements on plain radiographs [27,28,29]. They are also prone to significant errors due to potential angular deformities, limb girth differences, difficulties distinguishing bony landmarks, and differences in the relative positions of the limbs to the pelvis [28,30]. For these reasons, clinical methods are not recommended for most patients’ initial or serial LLD evaluation. Despite this, clinical techniques are still considered to be helpful when used as screening tools [31].

The most comprehensive way to evaluate most patients for leg length discrepancy (LLD) is using full-length anteroposterior (AP) computed radiography of both lower extremities while standing. The pelvis is typically leveled by placing a small lift under the shorter leg. However, full-length radiographs can be inconvenient due to the need for weight-bearing and the potential for magnification and parallax errors [23]. Before, during, and after any operations, AP pelvic radiographs, which include a view of the proximal femur, are the most convenient and commonly used for measuring LLD [32]. Other methods for determining LLD include CT, 3D ultrasonography, 3D biplanar low-dose X-ray devices (LDX), and magnetic resonance imaging (MRI). CT has the advantage of high inter-observer reliability compared to plain radiography; however, its use is limited due to cost, radiation exposure, and the inability to perform the scan with the patient standing erect [31,33].

Pelvic radiographs are often obtained pre-operatively for the general purpose of templating. Preoperative templating allows surgeons to plan and evaluate aspects of upcoming THA procedures. Not only does templating allow better prediction of the size of the prosthesis required, but it also aids in achieving appropriate offset and limb length equality and in minimizing intraoperative complications [34,35]. The general technique for assessing LLD on pelvic radiograph involves measuring the perpendicular distance between a bisecting line that passes through the lower edge of the teardrops or the ischial tuberosities to the tip of the lesser trochanters or the center of femoral heads. The perpendicular distance is measured on both sides, and the difference is the LLD [36]. Several studies have demonstrated that, depending on which pelvic and femoral landmarks are used for measurement, the resulting LLD can vary significantly and may be poorly reliable with poor intra- and inter-observer agreement [32,37]. Factors such as misalignment of pelvic positioning due to fixed flexion deformities, external rotation, and adduction/abduction contractures can also contribute to further reducing the precision and reliability of the LLD obtained by this method. Potential magnification errors introduced by the pelvic radiographs must also be considered [38]. Although there have been attempts to standardize patient positioning during X-rays to address these factors, the fundamental dependency of current LLD measurement methods on reference points that are based on two separate and independent segments (the pelvis and femur) produces a situation in which adjustment of leg position results in unwanted movement of the pelvis in the sagittal and coronal planes and vice-versa [38,39,40]. Variations in where the bisecting line is drawn for measurement on the pelvis and how surgeons choose given landmarks on the pelvis and femur further reduce the reliability of this measurement method. These variations are significant enough to cause false interpretations of the presence or absence of LLD [25,41].

This study aims to find the relationship between the most commonly used methods for evaluating leg length using pelvic X-rays and to compare how misalignment during X-rays affects leg length difference measurements. We also want to explore how effective it is to use trigonometric principles for leg length difference measurements. Our proposed technique only requires identifying a single anatomical landmark on each side. Unlike traditional methods, it allows for determining leg length differences regardless of the positions of the pelvis and femurs.

## 2. Methods

### 2.1. Participants

A total of 103 randomly selected patients (46 males, 57 females) who had undergone robotically assisted THA via MAKOplasty Total Hip Application between July 2012 and August 2014 were included in this study. These patients ranged in age from 43.8 to 79.1 years, with a mean of 67.6 and a mean BMI of 29.6 (SD = 5.3). All patients had the same acetabular prosthesis implanted, ranging from 42 to 60 mm and a median size of 54 mm. The acetabular component was an uncemented titanium porous-coated hemispherical cup, and the stem component was a Corrin Metafix Uncemented femoral stem (Trinity Corin, Cirencester, UK). Each of the 103 patients received a postoperative radiograph. We carefully excluded X-rays that needed to be more adequately centered (22.3%) as per standard guidelines and those with one or more bony landmarks that were not identifiable (6.87%) and would lead to additional errors. Additionally, all the X-rays used were meticulously adjusted in size based on the dimensions of the acetabular implants.

### 2.2. Radiological Measurement of LLD

**Figure 1:**
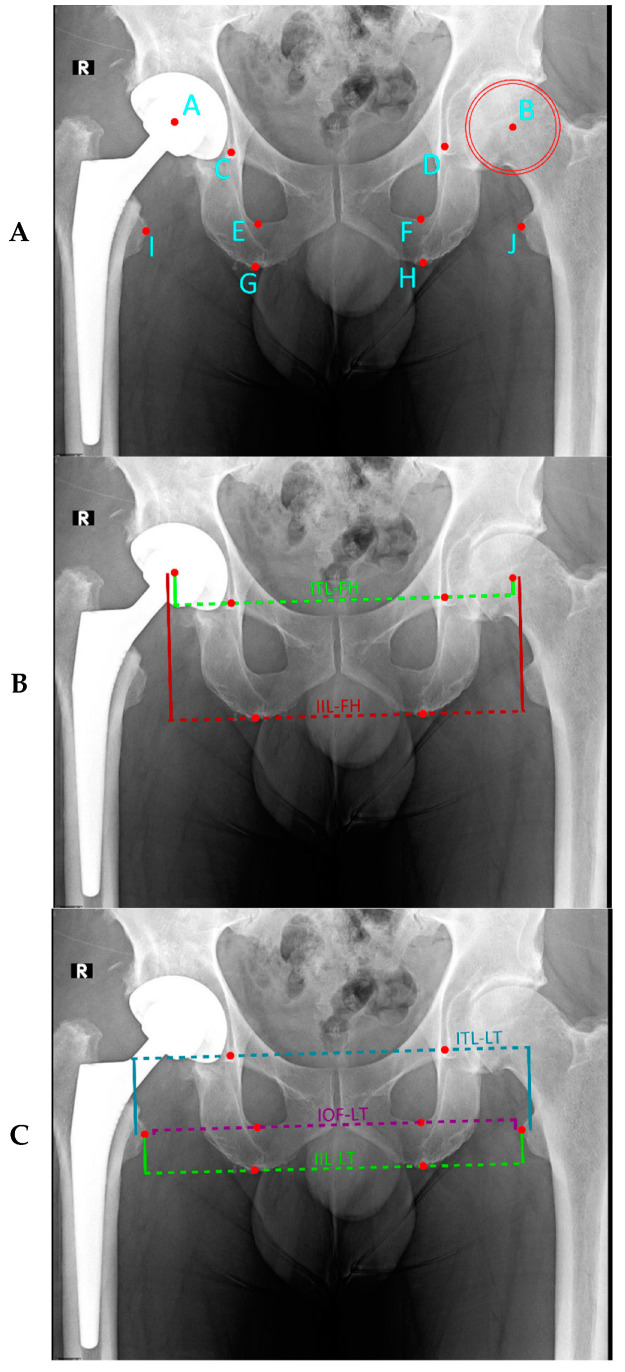
Measurements of LLD are detailed in Landmarks as denoted in Figure (**A**) by letters A–J (**A**), methods referenced to the femoral head (**B**), and methods referenced to the lesser trochanter (**C**).

Five measurement methods were chosen for this study based on their everyday use clinically and in other studies. We used three pelvic landmarks for bisecting lines (inter-teardrop line, inter-obturator foramina, inter-ischial line) and two femoral reference points (center of femoral head, lesser trochanter, Figure 1A). Measurements analyzed were the inter-teardrop line to the center of the femoral head (hereafter referred to as ITL-FH) [42,43] the inter-teardrop line to lesser trochanter (ITL-LT) [28,44,45], the inter-ischial line to the femoral head (IIL-FH) [46] the inter-ischial line to lesser trochanter (IIL-LT) [36,47,48], and inter-obturator foramina to lesser trochanter (IOF-LT) [38,49,50]. We measured LLD on postoperative AP pelvic radiographs using these five methods, each with its landmarks (Figure 1A–C). For prosthetic leg cases, femoral head centers were evaluated using the circle theorem [51]. Healthy femoral heads were reviewed using the Mose template method [52,53] (Figure 1A).

Obtained values were plotted, and linear regression was used to identify the relationship between measurement methods. During the final evaluation, the correlation matrix across methods was also conducted. For purposes of comparing the reported rates, we considered 10 mm to be the threshold for clinically significant LLD based on FEM and clinical studies [54,55].

### 2.3. Modeling Patient Misalignment

**Figure 2:**
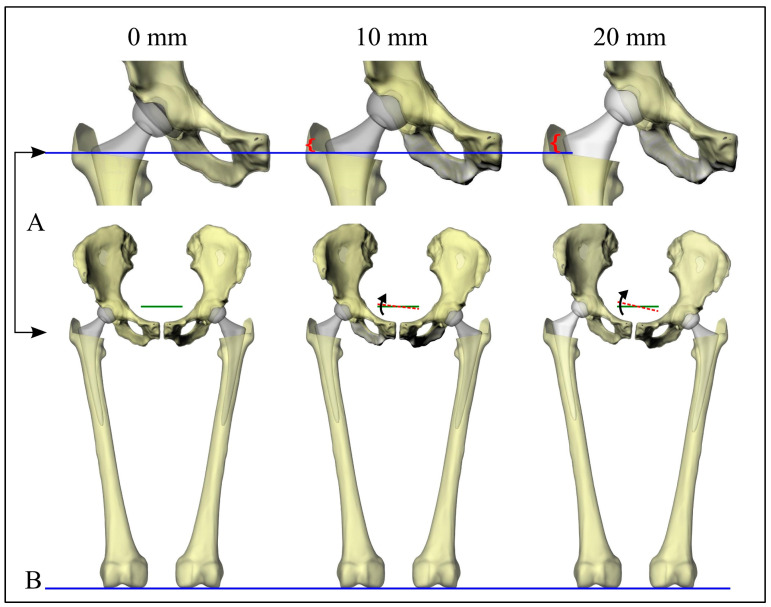
The variation in stem position for the three imposed LLDs (**A**) and its effect on the static limbs’ posture (**B**).

A computer model simulates joint movement at 3 imposed LLD values: 0, 10, and 20 mm. Motion in the sagittal, coronal, and transverse planes was modeled for both the femurs and the pelvis. This produced 9 movements that could be studied at each imposed LLD value, giving rise to 27 postures. For each posture, LLD was measured using the five previously described methods. Results were compared using Taguchi analysis [56], which uses an orthogonal array to identify which rotations most strongly affect measurements quickly. To obtain measurements not affected by the imaging technique adopted or by user bias in landmark selection, we developed a computer algorithm in the scripting language of Rhinoceros 3D (Robert McNeel & Associates, Seattle, WA, USA). This allowed us to simulate radiographs of given limbs, identify the landmarks used, and measure the LLD in a manner consistent with clinically used methods. Limb models were based on 3D bone geometry reconstructions produced using Materialise Mimics (Materialise, Leuven, Belgium). CT data were obtained using a BrightSpeed scanner (GE Medical Systems; slice thickness of 0.625 mm, pixel size of 0.422 mm). We used prosthetic components bilaterally to construct the computer model to remove any potential variability. Imposed LLD values were measured as differences in the height of the femoral head centers. They, therefore, gave rise to varying positions of the femoral stems (Figure 2A). The static standing posture resulted in pelvic coronal rotations of 0°, 3.12°, and 6.24° for the three cases considered (Figure 2B). 

**Figure 3:**
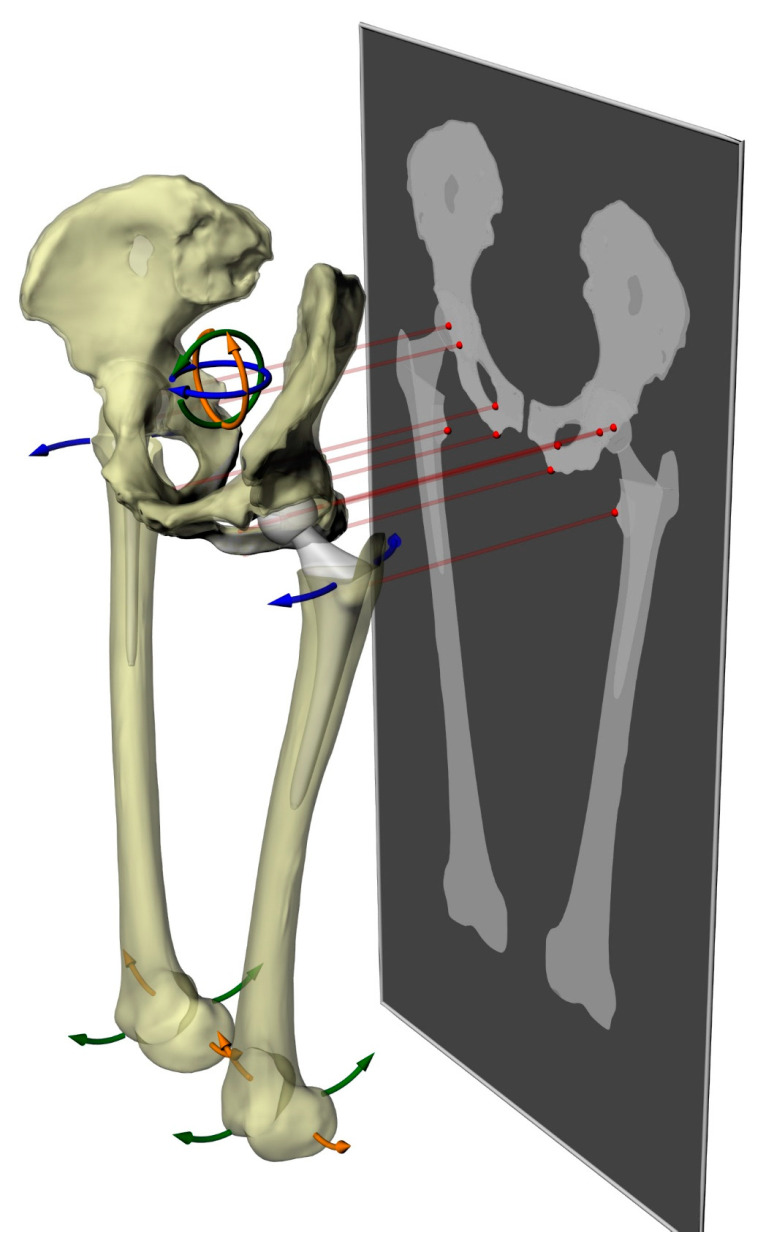
Illustration of the nine joint movements considered while projecting the limb geometry to simulate the X-rays and measure the LLD with the five analyzed measurement methods. Femoral movements (3 in each femur for a total of 6) considered were hip extension/flexion (green), hip abduction (orange, and hip external/internal rotation (blue). Pelvic movements (3 total) considered were anteversion/retroversion (green), left/right lateral rotation (orange), and axial rotation (blue).

Radiograph simulations were obtained by projecting the lower limbs on a coronal plane, representing the imaging plane, for 27 postures (Figure 3). To simplify the computations, the geometries were converted into triangular meshes, and the vertices were projected onto the imaging plane. The convex hulls of the points associated with each anatomical structure were then used to extract the landmarks employed for measurements. We adopted these settings to ensure that the only factor affecting variation in LLD measurements was the geometric orientation of the skeletal structures. To identify the influence of each joint movement on the measured LLD, we calculated the difference between the minimal and the maximal LLD values measured in the context of each movement and each method. The five methods of measurement were then correlated with the highest measurement differences found between rotation levels 1 (−2.5°), 2 (0°), and 3 (2.5°) and the joint movements that produced the respective differences.

### 2.4. Investigation of Methods Using Trigonometric Principles

**Figure 4:**
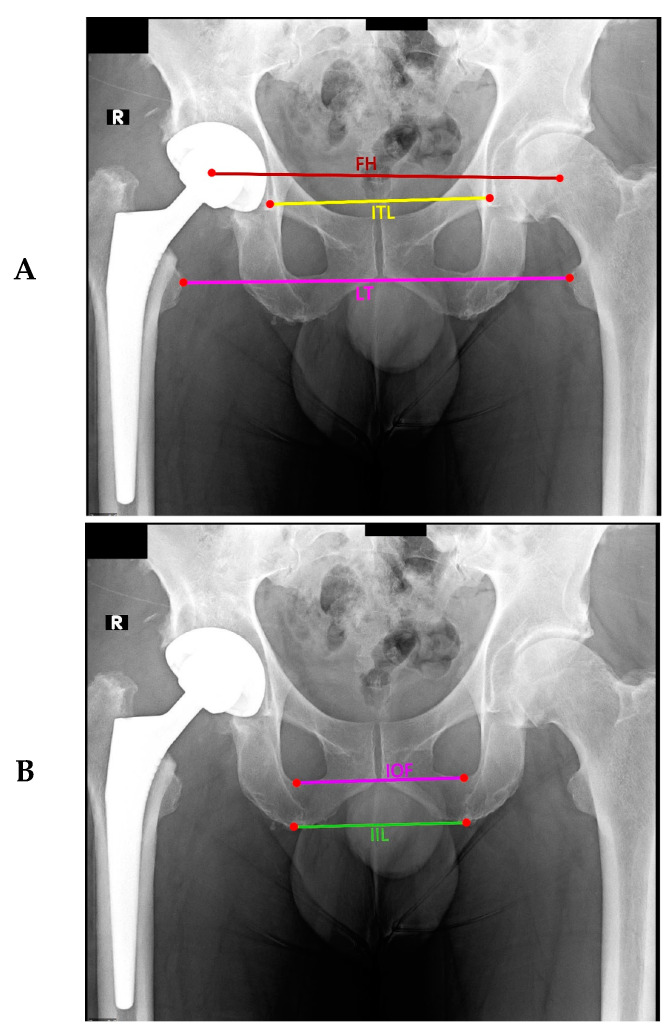
Measurements of LLD using trigonometric methods in which LLD is calculated as the product of the distance between the landmarks (the length of the connecting line) and the sine of the angle formed by the connecting line with the horizontal line. In (**A**) are illustrated the lines of Ischial teardrops (ITL), lesser trochanters (LT), and femoral heads (FH), while in (**B**) are shown the inter-ischial line (IIL) and inter-obturator foramen (IOF).

This method draws a line connecting an anatomical landmark on the left pelvis or femur to its partner on the right. A horizontal line concerning the radiograph is then drawn that intersects with the connecting line. The corresponding angle is trigonometrically measured and used to calculate the LLD, defined as the product of the distance between the landmarks (the length of the connecting line) and the sine of the angle (see Figure 4). As performed previously, LLD was measured using this and each previously described method, and linear regression was used to analyze the correlation between methods.

### 2.5. Statistics

The authors (MH, FA) independently measured LLD on all postoperative AP pelvic radiographs. To assess equivalence between the various measurement techniques, linear correlation was conducted using Microsoft Excel Data Analysis (Microsoft Office 2013; Microsoft Inc., Redmond, WA, USA). Inter-observer variability was evaluated using a paired *t*-test of measurements taken on a smaller random sample of 20 patients at a significance level of 0.05. In addition to Taguchi analysis, as described by Capetti et al. 2016 [56], single-way ANOVA was used to determine the equivalence between methods used for measuring LLD on simulated radiographs obtained via computer modeling.

### 2.6. Inter-Observer Variability

On a subset of radiographs, authors (MH, FA) measured LLD using the five previously described methods to evaluate their reliability. No significant difference between the two users was found. The measurement method utilizing the reference between the lesser trochanter and inter-ischial line (IIL-LT) resulted in the highest correlation of 0.94 (Table 1).

## 3. Results

### 3.1. Correlation between Current Methods

All conventional methods used to measure LLD on postoperative radiographs produced relatively similar averages with standard deviation taken into account. However, when LLD measurements were stratified based on a clinical significance of greater than 10 mm, methods varied in the percentage of cases they captured. Regarding femoral landmarks, methods that referenced the femoral heads reported at least 13.11% of cases to have clinically significant LLD, while methods that referenced the lesser trochanters reported at least 3.28% of cases to be clinically significant. Regarding pelvic landmarks, references to the inter-teardrop line reported at least 3.33% of cases, those to the inter-ischial line reported at least 9.84% of cases, and those to the inter-obturator foramina reported at least 3.28% of the cases to be clinically significant (Table 2).

**Figure 5:**
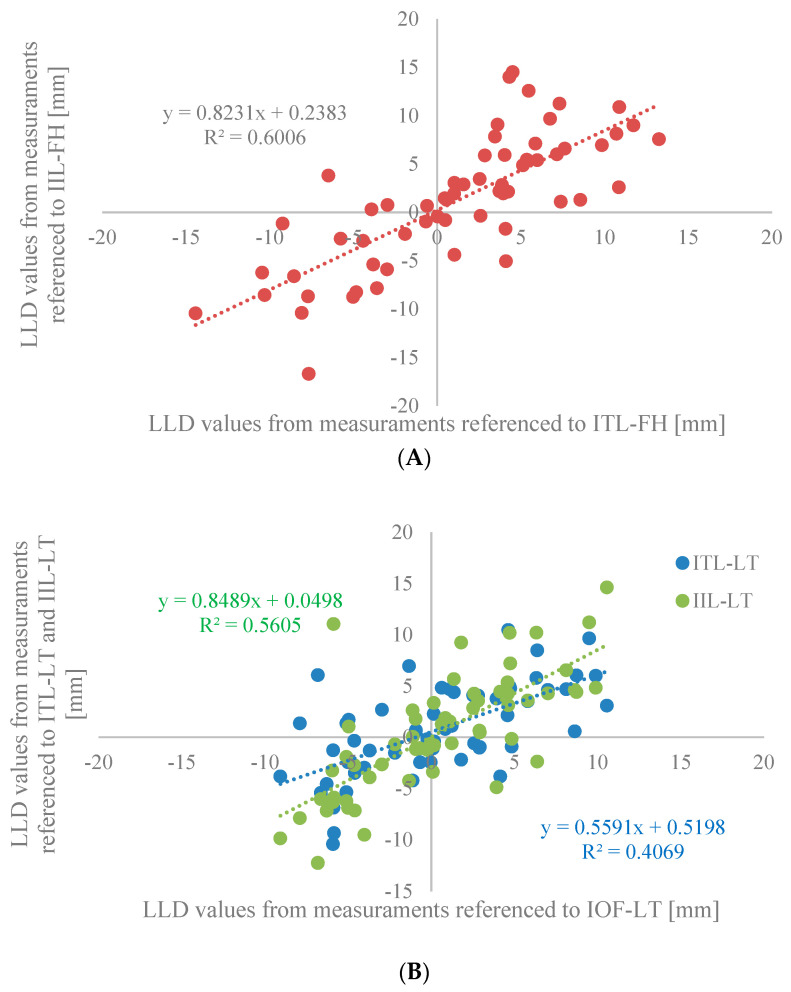
Measurements of LLD using (**A**) the methods referenced to the femoral heads center and (**B**) the three methods referenced to the lesser trochanters are shown.

Discordance between LLD measurement techniques is also demonstrated when the correlation between methods is evaluated (Table 3) and represented graphically (Figure 5A,B). A correlation coefficient of 0.74 was found between the two methods, which referenced the femoral heads (Figure 5A). Comparing methods that referenced the lesser trochanter (Figure 5B), the correlation between use of the inter-teardrop line relative to the inter-obturator foramina was 0.55, while the correlation between use of the inter-ischial line relative to the inter-obturator foramina was 0.65. Comparing methods that referenced the inter-ischial line, the correlation between the use of the femoral heads relative to the lesser trochanter was 0.38. Correlation between all other combinations of methods was found to be relatively poor.

### 3.2. Effect of Misalignment

Through our computer model, we imposed three levels of rotation (−2.5°, 0°, and 2.5°) on each of the 9 allowable planes of movement for the femoral and pelvic joints considered (Figure 3). This produced a total of 27 variable posture configurations through which we could produce limb length discrepancies of 0, 10, and 20 mm. After the projection of the 3D model onto a simulated 2-D radiograph, LLD was measured by each method. Average LLD values for each method are reported in Table 4.

**Figure 6:**
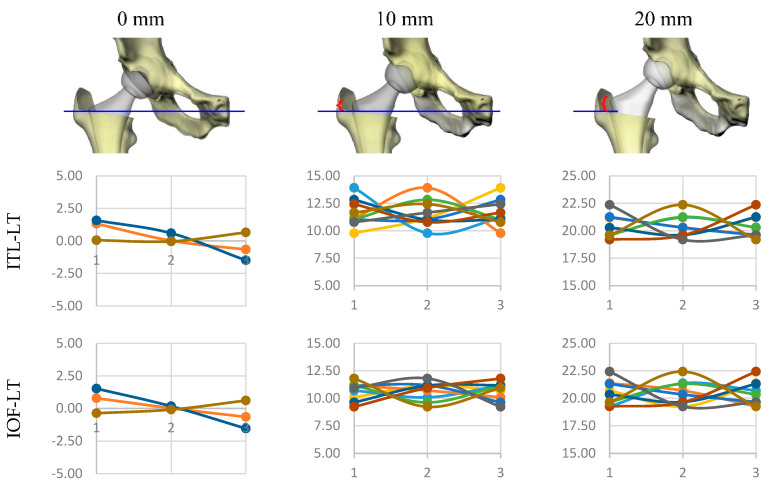

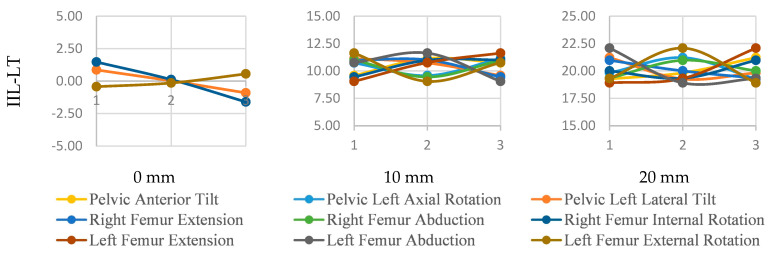
Taguchi’s analysis of the methods referenced to the lesser trochanters is shown. Each graph shows the measures of LLD obtained for each considered level (level 1 = −2.5°, level 2 = 0°, and level 3 = 2.5°) of each joint’s movement (color-coded).

It must be noted that both methods that referenced the femoral heads were unable to detect any variation in LLD in light of the hypothesis that the right cup was centered with the natural joint center. The other methods did not show significant differences in measured values, with the average measured LLD closest to the imposed LLD coming from the method referencing the inter-ischial line and lesser trochanter (IIL-LT). For each method and each imposed LLD in the model (0, 10, and 20 mm), we grouped the measured values of LLD for each considered level of each joint’s movement (level 1 = −2.5°, level 2 = 0°, and level 3 = 2.5°) and calculated the average (Figure 6).

Of the methods referenced to the lesser trochanters, those based on the ischium and obturator foramina gave similar results and are most affected by leg movements. In contrast, the method based on the inter-teardrop line is most sensitive to movements of both the leg and pelvis (Table 5). When comparing results between IOF-LT and IIL-LT that were not identical, we found the method based on the inter-ischial line (IIL-LT) is slightly more sensitive to pelvic tilt when compared to the method based on the obturator foramina (IOF-LT) for values of LLD greater than 10 mm (1.56 vs. 1.08 mm difference).

### 3.3. Trigonometric Methods

All five trigonometric methods used to measure LLD on postoperative radiographs resulted in similar average LLD values and standard deviations. However, stratification of measurements based on a clinically significant LLD of greater than 10 mm revealed variation in the percentage of cases captured by each method. The method which used the femoral head as its primary reference reported the largest percentage of clinically significant cases, 27.69%. The method referenced to the lesser trochanter reported 16.92% of cases as clinically significant, while the method referenced to the inter-ischial line reported only 1.54% of cases. Both the methods referenced the inter-teardrop line, and the inter-obturator foramina reported no cases with an LLD value more significant than 10 mm (Table 6).

**Figure 7:**
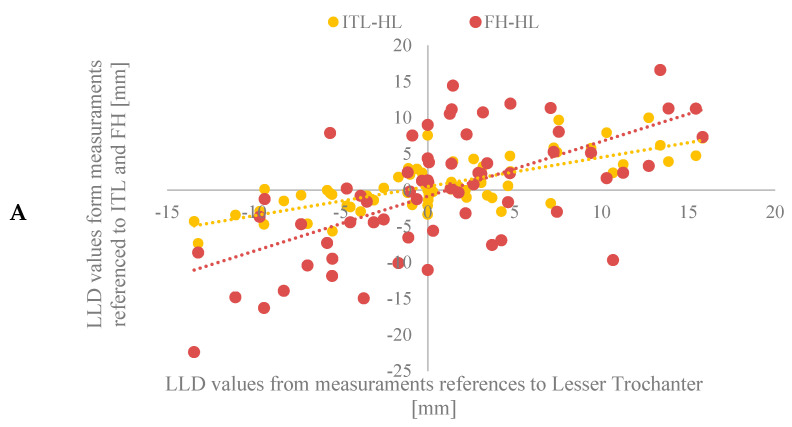

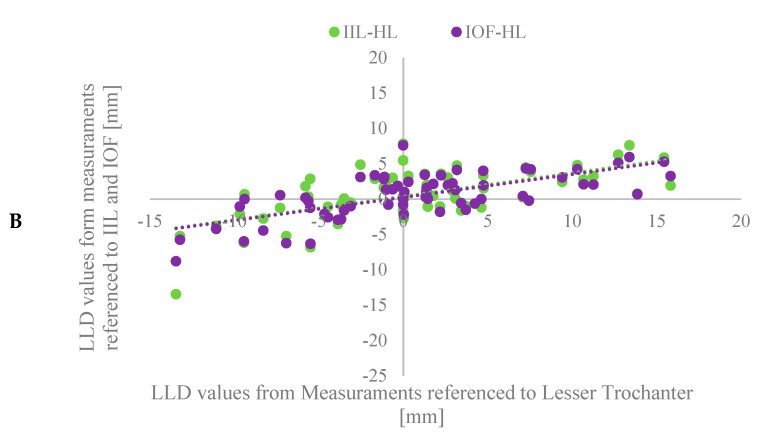
Measurements of LLD using trigonometric methods are shown for (**A**) the lesser trochanter method related to inter-teardrop line and femoral head methods and (**B**) the lesser trochanter method related to inter-ischial line and inter-obturator foramina methods.

The degree of correspondence between the various trigonometric measurement techniques is revealed when the correlation between methods is evaluated (Table 7) and represented graphically (Figure 7A,B). All ten comparisons of the five trigonometric-based methods were found to correlate greater than 0.60. In contrast, only two of the ten pairs of conventional methods compared demonstrated correlation values greater than 0.60 (Table 3).

## 4. Discussion

Considering the variability of methods used for measuring LLD and the potentially discordant results this could yield in clinical and research settings [57], this study sought to investigate the reliability and correlation between conventional methods to highlight the necessity for more uniformity within the orthopedic community. We also endeavored to determine the effects of changes in pelvic and femoral positioning on LLD measurements and propose an alternative method with advantages over current techniques. 

In comparing techniques, we found that certain methods were more reliably reproducible than others. Evaluation of inter-observer variability demonstrated that the most reliable methods were those that referenced the inter-ischial line (IIL-LT) or inter-obturator foramina (IOF-LT) to the lesser trochanter. Methods that referenced the femoral heads were substantially reliable but less reliable than those mentioned above. The use of the inter-teardrop line (ITL-LT) was found to be the least reliable method. 

Evaluating equivalence between conventional methods revealed that most correlated poorly with one another. Considering 10 mm as the threshold for clinical significance, we found that each method resulted in significantly different reporting rates for a percentage of captured cases. Reporting rates ranged from 3.28% (IOF-LT) to 14.75% (ITL-FH). Linear regression analysis further illustrated the lack of correlation between methods. 

When considering the effects of misalignment on LLD measurement, we found that certain methods were more sensitive to changes in limb positioning. In contrast, others were more sensitive to changes in pelvic tilt. Two methods referencing the lesser trochanter, one to the inter-ischial line (IIL-LT) and the other to the inter-obturator foramina (IOF-LT), were superior in minimizing errors related to postural changes. When subjected to alterations in pelvic and femoral alignment, these two methods resulted in similar patterns of variability. They were sensitive enough to detect LLD variations originating from acetabular and femoral components. On the other hand, the method that referenced the inter-teardrop line to the lesser trochanter (ITL-LT) was found to be overly sensitive to changes in pelvic rotation—demonstrating poorly accurate LDD values with high variability when subject to postural changes. Regarding techniques that used the center of the femoral heads as the femoral reference, we found they could not detect LLD variations unless the discrepancies being evaluated were a direct result of alterations in the alignment of the acetabular cup. We hypothesize that this finding may not necessarily reflect the innate inadequacy of the respective measuring techniques. Still, we may instead reflect the limitations of the computer model used in our study. Our model imposed pre-defined LLD values (0, 10, 20 mm) on variations in three-dimensional posture through joint movement in the sagittal, coronal, and transverse planes. In doing so, the model may not have had to significantly alter the positions of the acetabular cup prostheses to produce the pre-defined LLD or may not have altered cup positions in a plane discernable on the simulated radiographs. By not doing so, the acetabular cup prostheses remain in a state that too closely resembles the natural anatomical centers of the joints when projected onto 2D radiographs. Ultimately, the conventional methods that reference the femoral heads to measure LLD were not sensitive enough to detect these 3-D alterations on the simulated 2-D radiographs. 

Using trigonometric techniques to measure LLD, we found a high correlation between reported values, regardless of the femoral or pelvic landmark chosen for reference. However, as seen with conventional methods, trigonometric techniques varied in their ability to identify clinically significant LLD depending on the landmark used for measurement. Compared to traditional methods, which require the identification of two landmarks on each side, the trigonometric method requires only a single landmark on each side to be identified. This yields a practical advantage by eliminating potential sources of variation in landmark selection. Despite this, the trigonometric technique does have limitations. The most significant is that the method should ideally be used to measure LLD on standing, not supine, AP pelvic radiographs. This is due to dependence on an adequately aligned horizontal plane. Lateral pelvic tilt, caused by patients shifting while lying supine, could alter the positions of the reference points and lead to inaccurate measurements. Similarly, using this method on radiographs produced by improperly aligned imaging devices would also produce false measurements.

Implications of our findings challenge assertions made by previous studies. For instance, in assessing the variability of LLD measurements on plain radiographs, Kjellberg et al. [36] concluded that the method that referenced the inter-teardrop line to the lesser trochanter (ITL-LT) had excellent inter-observer reliability [36]. We found this method to be the least reliable of all conventional techniques. This discordance between our results may partially be explained by differences in sample size, statistical analysis techniques, and the fact that Kjellberg et al. [36] did not compare the abovementioned technique to any other conventional measurement method.

A study by Meermans et al. [37] compared LLD measured by the same conventional methods on preoperative AP pelvic radiographs with LLD values obtained from corresponding AP full-length radiographs. After analyzing the correlation of the average LLDs obtained from each set of corresponding radiographs, they concluded that methods that referenced the inter-teardrop line were more accurate than those that referenced the inter-ischial line. Regarding femoral landmarks used, they also concluded that methods referencing the femoral heads were more reliably reproducible than those referencing the lesser trochanters [37]. Our study findings dispute both these conclusions. We found that methods that referenced the inter-ischial line (IIL-LT) or inter-obturator foramina (IOF-LT) to the lesser trochanter were the most accurate. This was due to their superiority in minimizing errors related to pelvic and limb mispositioning and a higher sensitivity in quantifying LLD originating from acetabular and femoral components. Furthermore, although we concede that methods that referenced the femoral head centers (ITL-FH and IIL-FH) had significant inter-observer reliability—our results demonstrated that methods referencing the inter-ischial line (IIL-LT) or inter-obturator foramina (IOF-LT) to the lesser trochanter were still marginally more reliable. The disparities between our study results may be partially attributed to the different means by which we obtained the ‘true’ LLD valves used as a reference for assessing the accuracy and reliability of conventional methods. While we used a computer model to impose a precise LLD value as determined by computations in 3D space, Meermans et al. [37] obtained their values using the less accurate method of measuring LLD from hip center to ankle center on AP full-length radiographs. Other factors contributing to these disparities may include differences in statistical methods, and our study was performed using postoperative, not preoperative, radiographs. Additionally, unlike our study, Meermans et al. [37] did not consider the effects of underlying pelvic tilt on LLD—choosing to exclude patients who exhibited pelvic tilt related to soft-tissue contractures or spinal deformities for their study.

Another study by Heaver et al. [38] assessed the intra- and inter-observer reliability of various LLD measurement techniques on postoperative AP pelvic radiographs. Using radiographs of a synthetic pelvis and femur, they investigated the effects of pelvic positioning on LLD variability. The methods considered in their study included those referencing four pelvic landmarks (inter-teardrop line, inter-ischial line, inter-obturator foramina, and inferior sacroiliac joint) and two femoral landmarks (medial point of the lesser trochanter and tip of the greater trochanter). They concluded that measurements that referenced the inter-ischial line to the lesser trochanter (IIL-LT) were most reliably reproducible and least distorted by pelvic positioning [38]. Our results merit these findings as they corroborate that this method (IIL-LT) is highly reliable and less affected by pelvic mispositioning compared to conventional methods. However, unlike Heaver et al. [38], we found that the technique referencing the inter-obturator foramina and the lesser trochanter (IOF-LT) was nearly as reliable and unaffected by changes in pelvic alignment as the IIL-LT method. Heaver et al. [38] did not assess references to the center of the femoral heads. Therefore, no comparisons to our results can be made in this regard. 

Some authors have stated that the use of the inter-teardrop line should be preferred to the inter-ischial line, claiming that the inter-ischial line is generally an inferior landmark [36,37,45,58,59]. This conflicts with the findings of our study. Upon reviewing the citations used in these publications, we found a chain of references originating from a single study conducted by Goodman et al. [60], which found the ilio-ischial line, not the inter-ischial line, to be too poorly defined for reliable detection and measurement of acetabular migration on AP roentgenography [60]. In light of this, we propose using the inter-ischial line as a well-demarcated pelvic landmark.

We need to address some limitations of this study constructively. First, we only used a small number of radiographs (n = 73), but the high inter-observer reliability seen in the methods we used (refer to Table 1) supports our approach. It is important to note that similar limitations were seen in previous studies like Kuroda et al.’s survey, which was based on 48 cases [44], Meermans et al. [37]’s study, which involved 52 cases, and Edeen et al. [17]’s study, which included 68 cases. In all these cases, additional data would improve observer reliability and further demonstrate how errors in identifying landmarks can affect the results.

Second, this study’s scope was confined to robotic-assisted THA cases only. Regardless, our findings of poor correlation between measurement methods remain applicable to other THA surgical techniques.

One of our co-authors has demonstrated that robotic-assisted THA procedures are highly reliable in facilitating proper acetabular cup placement [61]. Others have shown that postoperative LLD after robotic-assisted THA is lower and less variable than manually performed THA [62]. In light of these advantages and the increasing popularity of robotic-assisted THA in clinical and research use [63], we consider the confines of this procedure to be an appropriate scope for this study.

This study focused solely on analyzing LLD measurement variation resulting from patient misalignment during radiograph positioning while neglecting to consider other influential factors such as parallax and magnification errors.

## 5. Conclusions

To our knowledge, this is the first study to objectively evaluate LLD measurement methods using computer algorithms. We found no correlation between the five most commonly used conventional methods when applied to robotic-assisted THA. Comparing the effects of misalignment on measurement accuracy, we found that methods referencing either the inter-ischial line or the inter-obturator foramina to the lesser trochanter were equally valuable. Our results underscore the necessity for researchers and clinicians to specify the methods used to assess LLD. Applying trigonometric principles to LLD measurement was shown to be accurate and reliable but contingent on proper radiographic alignment.

## Figures and Tables

**Table 1:** Inter-observer variability for LLD measurements was obtained using five conventional methods.

Methods	*p*-Value	Correlation Coefficient
ITL-FH	0.41	0.81
IIL-FH	0.36	0.88
ITL-LT	0.069	0.49
IOF-LT	0.18	0.93
IIL-LT	0.81	0.94

**Table 2:** Values of LLD measured on the postoperative X-rays.

	ITL-FH	ITL-LT	IIL-FH	IIL-LT	IOF-LT
Average	1.49	0.66	1.24	0.24	0.59
SD	6.53	4.51	6.63	5.83	5.26
Min	−14.43	−10.42	−16.70	−12.25	−9.07
Max	16.09	10.47	14.50	14.61	12.15
Range	30.52	20.89	31.20	26.86	21.22
% above 10 mm of LLD	14.75	3.33	13.11	9.84	3.28

**Table 3:** Correlation between LLD measurement methods found for the postoperative X-rays.

	ITL-FH	ITL-LT	IIL-FH	IIL-LT	IOF-LT
ITL-FH	1				
ITL-LT	0.352675	1			
IIL-FH	0.734999	0.263693	1		
IIL-LT	−0.04346	0.407669	0.382902	1	
IOF-LT	−0.04344	0.559982	0.231673	0.647262	1

**Table 4:** Average values and standard deviations of LLD are evaluated in the computer model for each value of imposed LLD with the five proposed methods.

Methods	Average Measured LLD [mm]			SD of Measured LLD [mm]			ANOVA (*p*-Value)		
	0 mm	10 mm	20 mm	0 mm	10 mm	20 mm	0 mm	10 mm	20 mm
ITL-FH	−0.025	−0.025	0.019	0.046	0.046	0.098	0.026	0.670	0.111
IIL-FH	0.081	−0.007	0.294	0.121	0.123	0.477			
ITL-LT	0.227	11.622	20.385	1.648	2.803	1.800	0.989	0.966	0.920
IOF-LT	0.052	10.654	20.440	1.541	1.459	1.915			
IIL-LT	−0.005	10.484	20.105	1.605	1.548	1.874			

**Table 5:** The highest differences found in LLD measures are between the low and high values of the variables and their source.

Methods	Highest Difference Found between Measured LLD Values		Source of the Highest Difference Found			
	0 mm	10 mm	20 mm	0 mm	10 mm	20 mm
ITL-FH	0.09	0.00	0.22	Pelvic Anterior/Posterior Tilt	--	Pelvic Coronal and Axial Rotations
IIL-FH	0.24	0.28	0.98	Pelvic Anterior/Posterior Tilt/Axial Rotations	Pelvic Axial Rotation	Pelvic Axial Rotation
ITL-LT	3.07	4.13	3.17	Right Femur	Pelvic Axial Rotation	Left Femur
IOF-LT	3.07	2.58	3.17	Right Femur	Left Femur	Left Femur
IIL-LT	3.07	2.58	3.17	Right Femur	Left Femur	Left Femur

**Table 6:** Values of LLD measured on postoperative X-rays using trigonometric methods.

	ITL-HL	LT-HL	FH-HL	IIL-HL	IOF-HL
Average	0.81	0.76	−0.28	0.64	0.48
SD	3.66	6.79	8.28	3.60	3.20
Min	−7.36	−13.45	−22.40	−13.46	−8.78
Max	10.00	15.83	16.60	7.84	7.61
Range	17.35	29.28	39.00	21.30	16.39
% Above 10 mm of LLD	0	16.92	27.69	1.54	0

**Table 7:** Correlation between LLD measurement methods found for postoperative X-rays using trigonometric methods.

	ITL-HL	LT-HL	FH-HL	IIL-HL	IOF-HL
ITL-HL	1				
LT-HL	0.750603	1			
FH-HL	0.538861	0.62226	1		
IIL-HL	0.732884	0.625699	0.604618	1	
IOF-HL	0.735128	0.692586	0.673611	0.920264	1

## Data Availability

Data supporting reported results can be derived using outlined procedures. Current datasets analyzed during the study require access to X-rays and CT images that are not available to the public.
